# From Needle to Necrosis: A Case Report on Nicolau Syndrome

**DOI:** 10.7759/cureus.100623

**Published:** 2026-01-02

**Authors:** Ramya A, Ambigai SSK, Adikrishnan Swaminathan

**Affiliations:** 1 Dermatology, Venereology and Leprosy, Sri Ramachandra Institute of Higher Education and Research, Chennai, IND

**Keywords:** cutaneous necrosis, embolia cutis medicamentosa, iatrogenic injection-site injury, intramuscular injections, nicolau syndrome

## Abstract

Nicolau syndrome is a rare but dreaded complication that can occur after a routine injection. It causes pain followed by ischemia and necrosis of the area, sometimes leading to serious and potentially life-threatening complications. Various drugs have been attributed to causing this, but only after being administered via an injection. What begins as pain and discoloration of the skin over the injection site quickly takes a sinister turn to become ischemic, ultimately leading to tissue necrosis. As the practice of administering injections is a major part of general medical practice, it becomes crucial to understand the inciting factors and know the potential complications of this otherwise harmless procedure.

Here we report a 51-year-old daily wage worker who had pain followed by dark discoloration and ultimately necrosis and ulceration following an intramuscular injection of diclofenac. This case report elaborates on various causes of Nicolau syndrome, the clinical presentations and treatment options of this disorder.

## Introduction

Nicolau syndrome (NS), also known as Embolia cutis medicamentosa, is an uncommon but serious complication following an intramuscular injection [1]. It can lead to various degrees of tissue necrosis ranging from intense pain and skin ulceration to ischemic necrosis of the entire limb [2]. While the pathogenesis of the disease still remains to be discovered, arterial wall irritation and arterial occlusion seem to play a major part in causing the ischemia and necrosis which heals with a disfiguring scar [3-5]. Various drugs, including non-steroidal anti-inflammatory drugs (NSAIDs), antibiotics, and vitamins, all of which are commonly administered as intramuscular injections in day-to-day practice, are implicated in the etiology of NS [6,7]. Considering the popularity of injections as an easy and effective way to administer drugs and treat patients, awareness and recognition of NS becomes important to a dermatologist.

## Case presentation

Here we report a 51-year-old man, a daily wage worker by occupation, who came to the hospital with dark discoloration and ulceration of the skin over the gluteal region. The patient was apparently normal five days earlier, after which he was treated at a local hospital with an intramuscular injection of diclofenac for complaints of pain in both legs. He revealed a history of pain over the injection site that started two hours after the injection and progressed overnight to an intense throbbing type of pain. There was also a history of redness of skin over the injection site that progressed to a dark discoloration of the region, ultimately leading to peeling of the skin and development of painful erosion over the injection site, after which he came to our hospital for further management.

There was no history of topical irritant application or native medication use. The patient also had no history of fluid or pus-filled lesions, or a history of penetrating trauma or surgery before the onset of lesions. He had no complaints of similar cutaneous or mucosal lesions. There was no history of any drug allergies in the past. He gave a history of being treated multiple times for similar complaints with diclofenac intramuscular injections, following which he had no such complaints.

**Figure 1:**
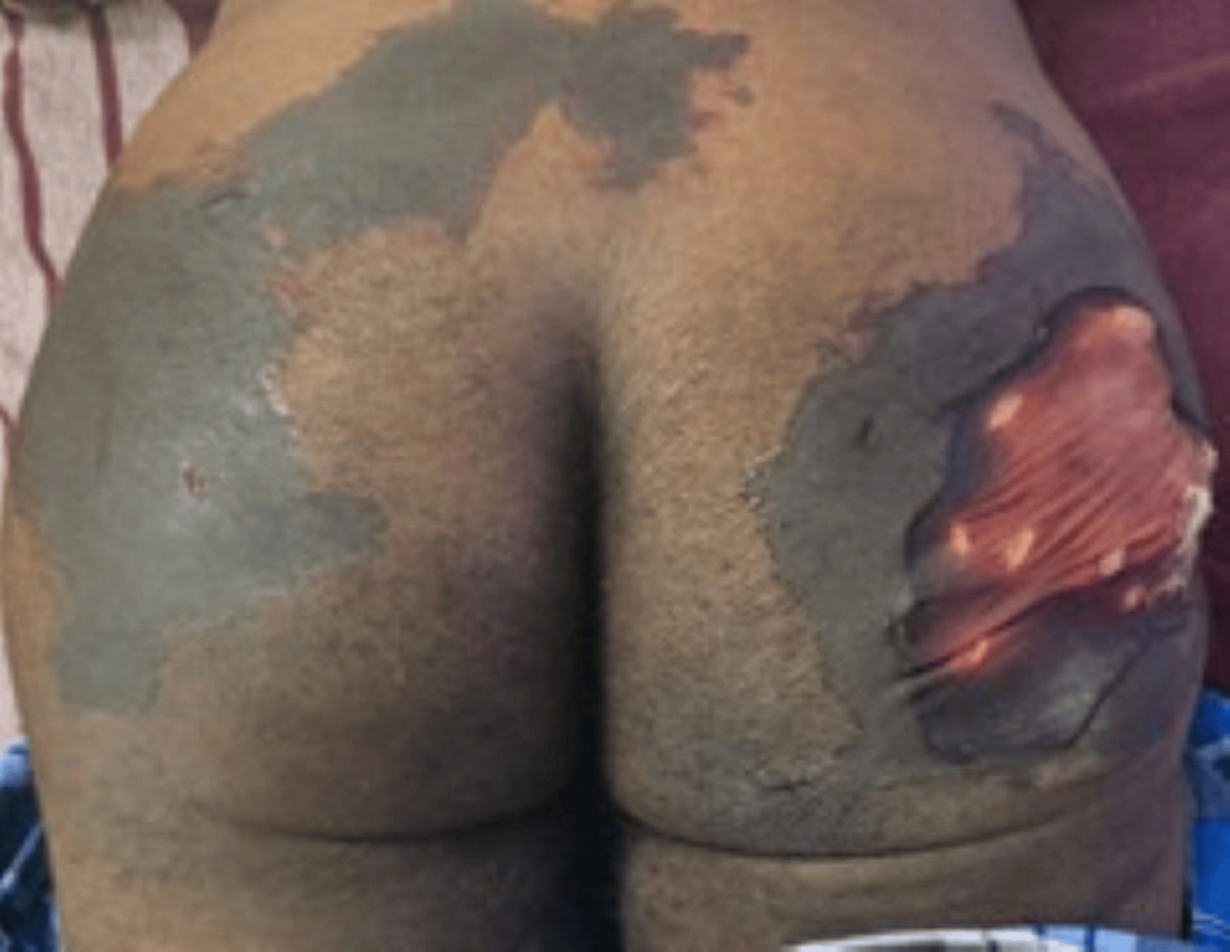
Areas of necrosis noted over the bilateral gluteal region

On examination, the vitals were normal and general examination was unremarkable with no lymphadenopathy. Dermatological examination showed a wide area of necrosis involving both bilateral gluteal regions of size 10 x 15 cm (Figure 1).

**Figure 2:**
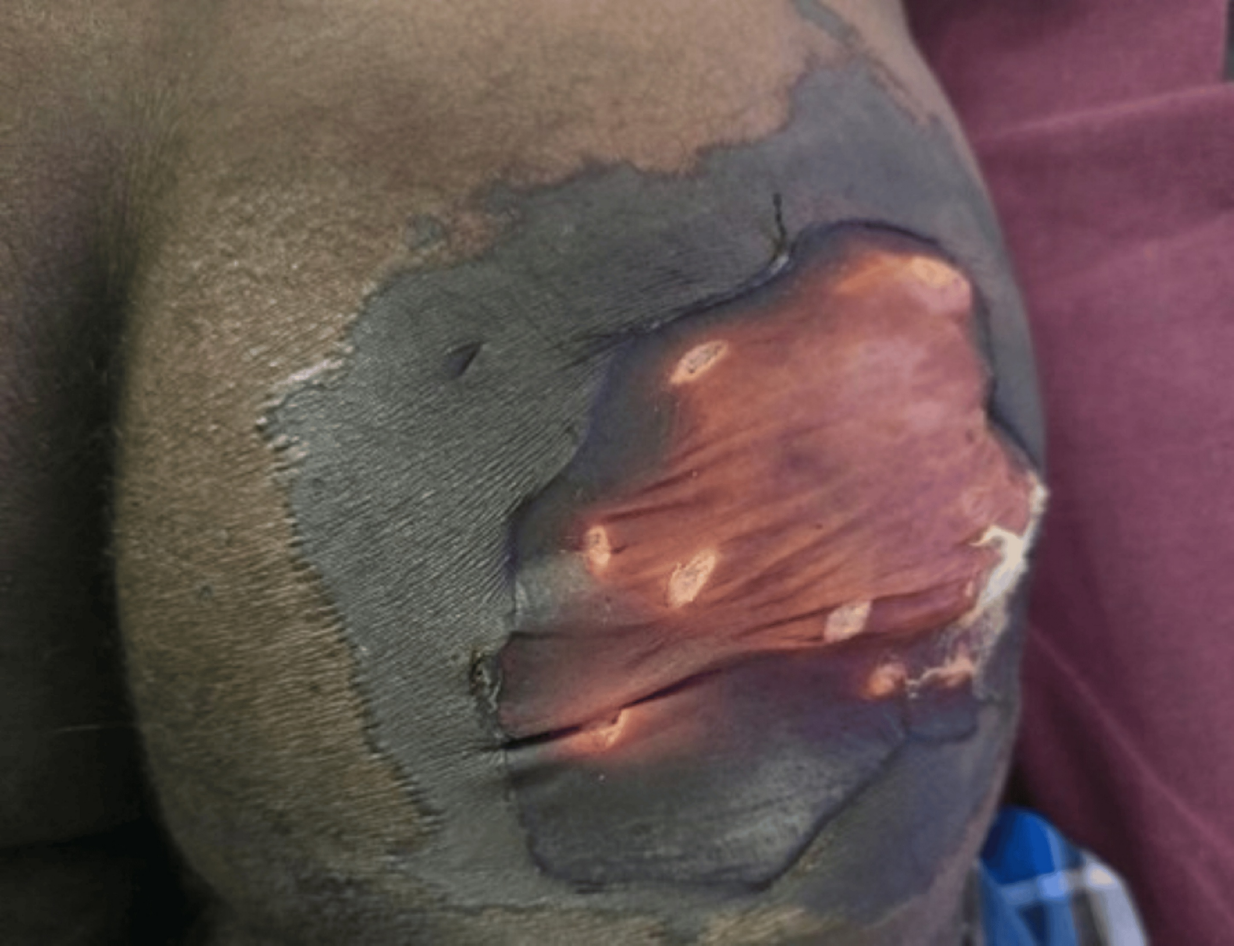
Area of ulceration noted over the right gluteal region

There was also a large erosion of size 6 x 5 cm noted over the right gluteal region (Figure 2).

Peripheral pulses of the bilateral lower limbs were normal.

The patient was admitted for further management. Though differential diagnoses like necrotizing fascitis and cellulitis were considered, after considering the arc of clinical history, a diagnosis of NS was made. He was started on broad spectrum IV antibiotics. Wound debridement was done and antibiotics with daily dressing were continued to which he responded well. The necrotic areas resolved with exfoliation followed by re-epithelialization. The patient was discharged with broad spectrum oral antibiotics and advice regarding proper wound care and dressing for the healing erosions and is currently under follow-up.

## Discussion

Described in the early 1920s by Nicolau and Freudenthal, NS was initially observed after the injection of bismuth salts for the treatment of syphilis [8,9]. It is an iatrogenic disease that can be caused by intra-arterial, intramuscular, intravenous, intra-articular or subcutaneous injections [8-13]. Various drugs, including NSAIDs, penicillin, steroids, Vitamin K, vaccines and dermatological procedures like hyaluronic acid fillers, mesotherapy and sclerotherapy, are attributed to causing NS [14,15].

The exact pathogenesis of NS is still being studied, but multiple theories have been offered. With the eventual outcome being ischemia and necrosis, vasospasm secondary to needle prick, embolus formation due to increased viscosity of the drug, and vasoconstriction due to periarticular injection are the various patho-mechanisms proposed [1]. Though multiple drugs are found to cause this syndrome, diclofenac, the drug that was administered to this patient, is the most common cause [1]. It is a cyclooxygenase inhibitor that inhibits prostaglandin synthesis, causing vasoconstriction, contributing to the pathogenesis of NS [16].

The site affected is based on the route of administering the injection, ranging from the thighs, arms, gluteal region, and the abdomen to the knees and shoulders. The three phases of the disease with clinical features and the various complications are detailed in Table 1.

**Table 1:** Clinical features of various stages of NS including the complications NS: Nicolau syndrome

Phase	Clinical features	Intervention	Reference
Initial phase Immediately after injection	Erythema to bluish discoloration of skin	External ice application worsens the extent of cutaneous necrosis and must be avoided at all costs	[1,17]
Acute phase lasts from 24 hours to 3 days post injection	Painful erythematous or livedoid indurated plaque; can also be called as livedoid dermatitis	Systemic steroids, anticoagulants	[1,18]
Necrotic phase from 5 to 15 days	Tissue necrosis leading to erosions and ulcers	Broad-spectrum antibiotics, analgesics, daily dressings, and surgical debridement	[1]
Immediate complications	Necrotizing fasciitis, sepsis, death due to septic shock	Broad-spectrum antibiotics, analgesics, daily dressings, and surgical debridement	[1]
Late complications	Extensive scarring, contractures, deformities,	Corrective surgeries	[1]
Rare sequelae	Soft tissue sarcoma	Biopsy	[1]

Clinical suspicion and relevant history taking play a major role in diagnosis as mentioned in this case report. Other findings noted in the various investigations are listed in Table 2.

**Table 2:** Findings noted in various investigations [1]

Investigations	Findings
Ultrasonography	Area of diffuse edema within the muscles
Computerized tomography	Well-defined lesion with inflammatory changes and central gas collection
Magnetic resonance imaging	Areas of focal muscle necrosis
Histopathological examination	Fat necrosis with predominant eosinophilic infiltration with no evidence of vasculitis

## Conclusions

Thus, NS is a rare yet distressing disorder of the skin that follows an injection. Though the intramuscular route was found to be most commonly associated, injections in general are found to be an inciting factor. The commonness of the causative factor, the excruciating pain faced by the patient, and the risk of sepsis and the disfigurement that follows healing, all make understanding and preventing this disease crucial. Though the pathogenesis is not fully understood, early identification plays a vital role in treating this disorder. Educating the general practitioners about the right principles of administering injections and explaining the perils should this disease occur is vital to reduce the incidence of this iatrogenic syndrome.
